# KMO Inhibition Improves Seizures and Depressive-like Behaviors Without Aggravating Cognitive Impairment in Epileptic Mice

**DOI:** 10.3390/cimb47090705

**Published:** 2025-09-01

**Authors:** Jingwen Xu, Yifen Huang, Liping Wei, Ziting Kong, Junling Fu, Lun Cai

**Affiliations:** 1 Department of Neurology, The First Affiliated Hospital of Guangxi University of Chinese Medicine, Guangxi University of Chinese Medicine, No. 89-9 Dongge Road, Nanning 530023, China; xujingwen2023@stu.gxtcmu.edu.cn (J.X.); yifenhuang2022@stu.gxtcmu.edu.cn (Y.H.); kongziting2024@stu.gxtcmu.edu.cn (Z.K.); fujunling2024@stu.gxtcmu.edu.cn (J.F.); 2 Guangxi Key Laboratory of Molecular Biology of Traditional Chinese Medicine and Preventive Medicine, Nanning 530023, China; 3 Department of Rehabilitation, The First Affiliated Hospital of Guangxi University of Chinese Medicine, Guangxi University of Chinese Medicine, Nanning 530023, China; 13978856423@163.com

**Keywords:** epilepsy, depression, kynurenine pathway, KMO, cognitive function, Ro 61-8048

## Abstract

The objective of this study is to investigate the effects of kynurenine-3-monooxygenase (KMO) inhibition on seizures, depressive-like behaviors, and cognitive functions in epileptic mice, and to elucidate its impact on the kynurenine metabolic pathway. Male Kunming (KM) mice were randomized into four groups: the epileptic model (EM), epileptic model treated with Ro 61-8048 (RM), healthy control (HC), and healthy control treated with Ro 61-8048 (RC). Chronic epilepsy was induced in the EM and RM groups via an intraperitoneal pilocarpine injection (225 mg/kg). The RM and RC groups received Ro 61-8048 (42 mg/kg). The seizure frequency was monitored continuously using a 24 h video recording. Depressive-like behaviors were assessed with the sucrose preference test (SPT) and forced swim test (FST); cognitive function was evaluated with the Y-maze test and open field test (OFT). The concentrations of kynurenine (KYN), kynurenic acid (KYNA), 3-hydroxykynurenine (3-HK), and 3-hydroxyanthranilic acid (3-HANA) were determined by liquid chromatography–tandem mass spectrometry (LC–MS/MS). Compared to the EM group, the RM group exhibited a reduced seizure frequency and severity (*p* < 0.05), ameliorated depressive-like behaviors (increased sucrose preference in SPT, and decreased immobility time in FST, *p* < 0.05), and enhanced cognitive performance (elevated spontaneous alternation and reduced non-sequential alternation in a Y-maze, and increased time and distance in a central open field area, *p* < 0.05). Mechanistically, compared to the RC group, the RM group showed an increased KYNA/KYN ratio, and a decreased 3-HK/KYN ratio (*p* < 0.05) KMO inhibition rectifies the neurotoxic–neuroprotective imbalance in the kynurenine pathway (downregulating the 3-HK/3-HANA ratio and upregulating the KYNA/KYN ratio), thereby decreasing seizures, depressive-like behaviors, and cognitive deficits. These findings suggest KMO inhibition is a potential therapeutic strategy for epilepsy-associated depression. A further investigation of its mechanisms and clinical applicability is warranted.

## 1. Introduction

Epilepsy, a neurological disorder characterized by highly synchronized abnormal neuronal discharges in the brain, affects approximately 3% of the global population [1]. Patients with epilepsy face a twofold increased risk of comorbid depressive disorders compared to the general population [2]. However, the use of conventional anti-depressant medications in these patients is frequently constrained due to the potential drug–drug interactions, limited efficacy, or increased seizure risk [3,4,5]. These limitations may significantly impair patients’ quality of life and elevate their suicide risk [6,7]. Therefore, investigating the pathogenesis of epilepsy-associated depression and developing novel, safe, and effective therapeutics is essential.

**Figure 1:**
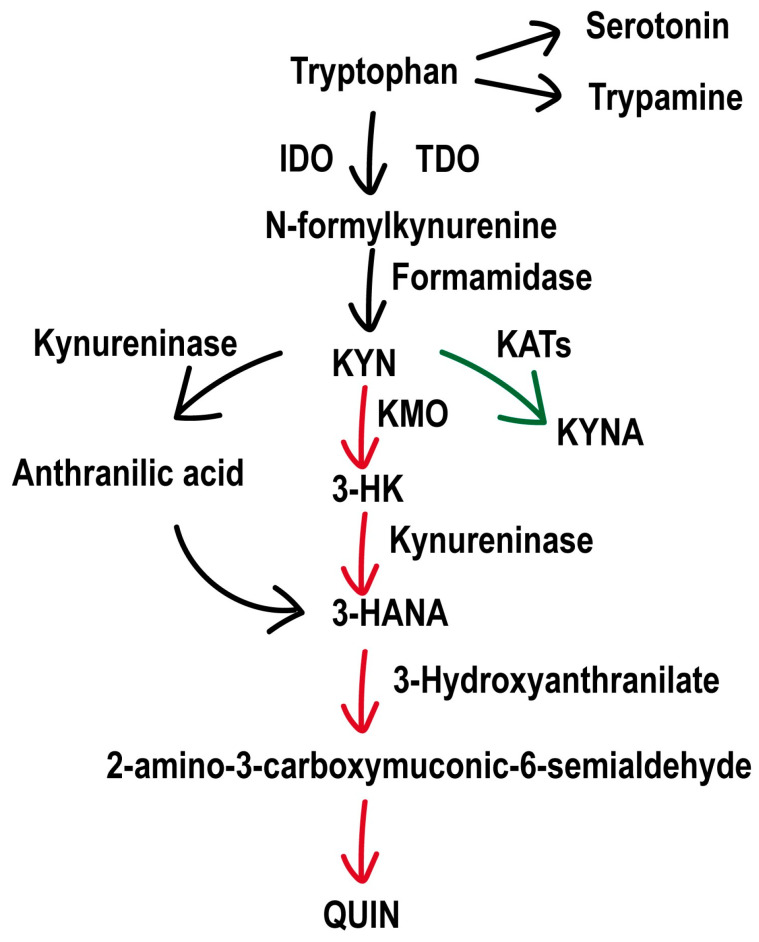
Tryptophan metabolic pathway. The schematic illustrates key enzymatic branching points. Tryptophan is initially converted to *N*-formylkynurenine by indoleamine 2,3-dioxygenase (IDO) or tryptophan 2,3-dioxygenase (TDO), then hydrolyzed to kynurenine (KYN) by formamidase. KYN undergoes KMO-mediated hydroxylation to form 3-hydroxykynurenine (3-HK), which is further metabolized to neurotoxic metabolites, including 3-hydroxyanthranilic acid (3-HANA) and quinolinic acid (QUIN). Alternatively, KYN is transaminated by kynurenine aminotransferases (KATs) to generate kynurenic acid (KYNA), conferring neuroprotection. A minor branch yields neurotransmitters such as serotonin and melatonin.

Recent studies have indicated that the neuroinflammation-mediated dysregulation of the tryptophan–kynurenine pathway may underlie the core pathology of epilepsy-associated depression. Recurrent epileptic seizures activate inflammatory mediators such as Interleukin-1 beta (IL-1β) and upregulate the expression of kynurenine 3-monooxygenase (KMO), a crucial enzyme in the kynurenine pathway [8]. This upregulation drives the metabolic flow toward the neurotoxic branch, resulting in the production of 3-hydroxykynurenine (3-HK), 3-hydroxy-o-cyanobenzoic acid (3-HANA), and quinolinic acid (QUIN) [9]. Figure 1 shows the tryptophan–kynurenine pathway, including the key enzymes, branches, and metabolites’ neurotoxic/neuroprotective effects. These neurotoxic metabolites induce direct neuronal damage through oxidative stress and *N*-methyl-D-aspartate (NMDA) receptor hyperactivation [10], while also disrupting dopaminergic [11,12] and serotonergic (5-HTergic) neurotransmission [13,14], ultimately leading to neurotransmitter imbalances and depressive-like phenotypes [15].

In the kynurenine pathway, KMO serves as a pivotal regulator of the branch equilibrium. Its overactivation diverts the kynurenine (KYN) flux toward neurotoxic metabolites: 3-HK induces oxidative stress via reactive oxygen species generation, potentially lowering seizure thresholds [16]; 3-HANA facilitates free radical formation, exacerbating inflammatory responses and epileptogenic QUIN production [17]; and QUIN acts as an NMDA receptor agonist, triggering neuronal excitotoxicity and apoptosis through glutamatergic hyperactivation [18]. These toxic products antagonize the neuroprotective branch metabolite kynurenic acid (KYNA): as an endogenous glycine-site NMDA receptor antagonist, KYNA counteracts glutamatergic overexcitation and exerts neuroprotection [19,20]. Paradoxically, aberrantly elevated KYNA may excessively inhibit neurotransmission, causing cognitive dysfunction [21,22,23], whereas reduced levels compromise their protection against QUIN toxicity [24], presenting a therapeutic challenge for KMO modulation.

Thus, when evaluating KYNA’s role in epilepsy-associated depression, balancing neuroprotection against the cognitive impairment risk is critical. In this study, we employed a murine epilepsy model treated with the KMO inhibitor Ro 61-8048, a selective inhibitor extensively validated preclinically that specifically binds and inhibits KMO enzymatic activity [25,26,27]. Using this approach, we investigated the effects of KMO inhibition on seizures, depressive-like behaviors, and cognitive function in epileptic mice, thereby providing novel insights and a robust scientific foundation for developing therapeutic strategies targeting epilepsy-associated depression.

## 2. Materials and Methods

### 2.1. Animals

**Figure 2:**
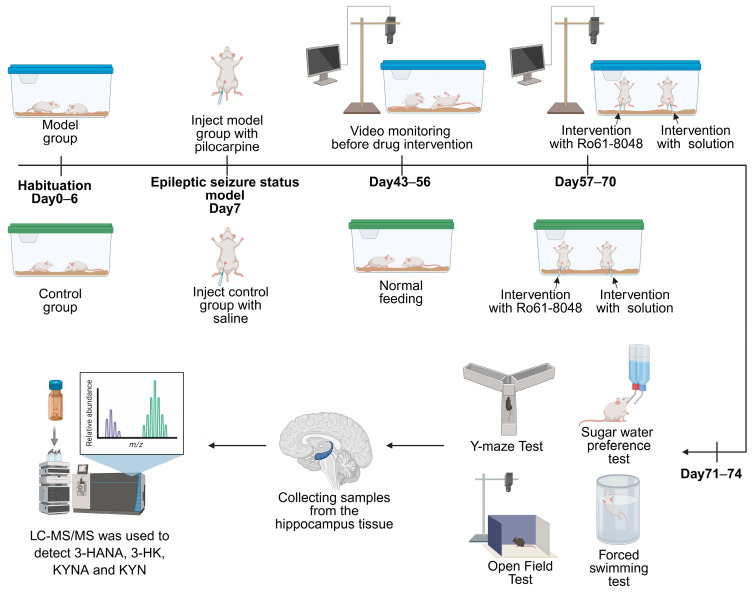
Experimental timeline of the murine epilepsy study. The study was conducted in phases. After a 6-day acclimatization period, the model group received pilocarpine on Day 7 to induce epileptic seizures, while controls received an equivalent volume of saline. Video monitoring for behavioral assessment was initiated on Day 43 and continued until Day 70. Interventions with Ro61-8048 or vehicle were administered from Day 57 to Day 70. Behavioral assessments using Y-maze, open field, and forced swim tests were performed from Day 71 to Day 74. Subsequently, hippocampal samples were analyzed for 3-HANA, 3-HK, KYNA, and KYN levels using LC-MS/MS. LC–MS/MS: liquid chromatography–tandem mass spectrometry.

Thirty-six male Kunming mice (5 weeks old, 30–34 g) were obtained from Hunan SJA Laboratory Animal Co., Ltd. (Changsha, China) (License No.: SCXK(Xiang)2019-0004). The mice were housed under controlled conditions (23 ± 2 °C, 50 ± 10% humidity) with a 12-h light/dark cycle. All procedures strictly adhered to institutional and national guidelines for the use and welfare of laboratory animals, approved by the Animal Ethics Committee of Guangxi University of Chinese Medicine (Approval Code: DW20240603-150; Approval Date: 3 June 2024; see Figure 2 for experimental timeline).

### 2.2. Establishment of Epilepsy Model Mice

Following a one-week acclimatization period, thirty-six 6-week-old healthy Kunming mice, weighing 35−43 g, were randomly assigned. Twenty-four mice were designated for the epilepsy model group. These mice received an initial intraperitoneal injection of *N*-methylscopolamine nitrate (2 mg/kg, Tokyo Chemical Industry, Tokyo, Japan) and terbutaline hemisulphate (2 mg/kg, 3ABIO, Shanghai, China). Thirty minutes later, they received an intraperitoneal injection of pilocarpine hydrochloride (225 mg/kg, Sigma-Aldrich, St. Louis, MO, USA). The remaining 12 mice served as the control group and received equivalent volumes of sterile saline instead of pilocarpine.

Post-injection, the mice were maintained at a constant temperature of 23 ± 2 °C. Seizure activity was monitored and assessed using the Racine grading criteria [28], defined as follows: stage 1, rhythmic clonic twitching of the perioral, auricular, or facial musculature; stage 2, nodding movements accompanied by facial clonus; stage 3, bilateral forelimb clonus without rearing; stage 4, bilateral forelimb clonus with rearing; and stage 5, generalized tonic–clonic convulsions culminating in loss of posture control. If no seizure occurred within 30 min or if seizure severity did not reach stage 4, a supplemental dose of one-third of the initial pilocarpine hydrochloride dose was administered intraperitoneally every 30 min, up to a maximum of three doses. After 1.5–2 h of continuous seizure activity, seizures were controlled by intraperitoneal injection of diazepam (10 mg/kg), followed by subcutaneous administration of 0.5 mL 5% dextrose solution and 0.5 mL 0.9% sodium chloride solution.

Mice failing to achieve Stage 4 seizures were considered modeling failures. Ultimately, 12 mice (50% of the 24 modeled) survived and were successfully modeled. These mice were subsequently randomly divided into two experimental groups: the epilepsy model group (EM; *n* = 6) and the Ro 61-8048 model group (RM; *n* = 6). Similarly, control mice were divided into the healthy control group (HC; *n* = 6) and the Ro 61-8048 control group (RC; *n* = 6).

### 2.3. Drug Preparation and Intervention

Ro 61-8048 powder (TargetMol, Shanghai, China) was dissolved in a vehicle solution composed of Tween 80 (Mreda, Beijing, China), dimethyl sulfoxide (DMSO; Beyotime, Beijing, China), polyethylene glycol 300 (PEG 300; HarveyBio, Beijing, China), and sterile water in a volume ratio of 1:2:6:11. A solution of Ro 61-8048 was prepared in this vehicle to deliver 42 mg/kg per injection. The RC and RM groups received intraperitoneal injections of the Ro 61-8048 solution (42 mg/kg) on experimental days 1, 2, 3, 4, 6, 8, 10, 12, and 14 of week 7 [29,30,31]. The HC and EM groups received volume-matched intraperitoneal injections of the vehicle solution (without Ro 61-8048) at identical time points.

## 3. Observational Indicators

### 3.1. Video Monitoring of Seizure Frequency and Severity

Beginning in the fifth week post-modeling, mice from all groups (n = 6 per group) were video-monitored using a video-monitoring system. This system recorded both seizure frequency and severity (assessed using the Racine scale). During analysis, seizure severity was quantified using the following scoring system: Stage 1 = 1 point; Stage 2 = 2 points; Stage 3 = 3 points; Stage 4 = 4 points; and Stage 5 = 5 points. Monitoring covered the intervention period (weeks 7 and 8), concluding at the end of week 8. This enabled a comparative analysis of seizure activity pre- and post-intervention.

### 3.2. Body Weight Tracking

Body weight was measured as a critical indicator of overall health status at key time points: baseline (pre-acclimation), subsequent post-acclimation (Day 7), 1-week post-modeling (Day 14), pre-intervention (Day 56), 1-week post-intervention (Day 63), and 2-weeks post-intervention (Day 70). These longitudinal measurements provided a continuous assessment of general health throughout the study.

### 3.3. Depression-like Behavioral Experiments

Sucrose Preference Test (SPT) to assess anhedonia in mice [32,33]: On day 1, mice from each group (*n* = 6) were individually housed in cages, each presented with two bottles containing 1% sucrose solution (100 g each) for 24 h to habituate. On day 2, each cage received one bottle of 1% sucrose solution and one bottle of plain water (100 g each) for another 24 h. On day 3, mice were deprived of food and water for 24 h. On day 4, mice remained food-deprived but were given access to one bottle of 1% sucrose solution and one bottle of plain water (100 g each). The total consumption of the sucrose solution and plain water was measured after 24 h. The percentage of sucrose water consumption was calculated using the following formula:

$$SucrosePreference(\%)=\frac{Sucrosesolutionconsumed(g)}{Totalliquidconsumed(g)}\times 100$$

Forced Swim Test (FST) to evaluate depression-like behavior in mice [34,35]: Mice were placed individually in a transparent plexiglass cylinder (30 cm high × 20 cm diameter) within a soundproof box (800 Lx). The cylinder contained water (23 ± 1 °C) to a depth of 25 cm. Each mouse was allowed to swim freely for 6 min. The immobility time, defined as passive floating with only minimal movements necessary to keep the head above water, was recorded during the final 5 min of the test. The percentage of time spent immobile was calculated as follows:

$$Immobility\;\left(\%\right)=\frac{Immobilitytime(s)}{Totaltestduration(s)}\times 100$$

### 3.4. Cognitive Function Assessment

Exploratory behavior, a key indicator of cognitive function [36,37], was assessed using the Open Field Test (OFT) on day 2 of week 9. Individual mice were placed in the center of an open field arena (50 cm × 50 cm × 40 cm). The floor was divided into 25 equal squares, with the central 9 squares defined as the center zone and the peripheral 16 squares as the periphery. The mice explored freely for 5 min. The total distance traveled (cm), center zone distance (cm), and center zone duration (s) were recorded via video tracking. The center time percentage and center distance percentage were calculated as follows:

$$CentralTime\;(\%)=\frac{Timeincenterzone(s)}{Totaltime(s)}\times 100$$

$$CentralRegionDistance(\%)=\frac{Distanceincenterzone(cm)}{Totaldistancetraveled(cm)}\times 100$$

Y-maze experiments were performed on days 3–4 of week 9 using a three-arm apparatus (arms: 30 cm L × 10 cm W × 15 cm H; 120° inter-arm angles) integrated with Visu Track software (Version 2.0, Shanghai XinSoft Information Technology Co., Ltd., Shanghai, China). The protocol comprised two consecutive phases: (1) training phase: mice explored freely from the central platform for 5 min; and (2) test phase: after a 10 min inter-trial interval in home cages, mice were reintroduced to the center for 5 min exploration with arm entry sequences recorded. The same-before alternation rate was calculated for incorrect sequential entries (e.g., AAB, AAC) using the following:

$$DisorderedAlternationRate(\%)=\frac{DisorderedAlternations}{Entries-2}\times 100$$

Twenty-four hours post-test, the spontaneous alternation rate was assessed for correct sequential entries (e.g., ABC and ACB):

$$GeneralAlternationRate\left(\%\right)=\frac{GeneralAlternations}{Entries-2}\times 100$$

### 3.5. Liquid Chromatography–Tandem Mass Spectrometry (LC–MS/MS) for the Quantification of KYN, KYNA, 3-HK, and 3-HANA Metabolites

Hippocampal tissues (50 mg) were homogenized in 480 μL methanol containing 20 μL internal standard working solution (250 ng/mL) using a cryogenic grinder at −20 °C. After 30 min of incubation, samples were centrifuged twice (14,000× *g*, 10 min, 4 °C). The supernatant (300 μL) was transferred to a new tube, dried under a nitrogen stream, and reconstituted in 100 μL methanol: water (1:9, *v*/*v*). Following centrifugation (14,000× *g*, 10 min), the final supernatant was transferred to LC vials. Chromatographic separation was performed using an Agilent 1290 Infinity II UHPLC system coupled to a 6470 triple quadrupole mass spectrometer (Agilent Technologies, Santa Clara, CA, USA).

Chromatographic conditions: mobile phase A: 0.1% formic acid; mobile phase B: methanol; flow rate: 0.2 mL/min; column temperature: 25 °C; injection volume: 2 μL; gradient elution: 0~3 min, 0% B; 0~3 min, 0%~100% B; and 10~12 min, 100%~0% B. The 10~12 min effluent was switched to the waste liquid and did not enter into mass spectrometry detection.

Mass spectrometric detection was conducted in positive electrospray ionization (ESI+) mode with multiple reaction monitoring (MRM). Key parameters: sheath gas heater: 250 °C, sheath gas flow: 12 L/min, V charging: 500 V, nebulizer: 40 psi, capillary: 2500 V, gas flow: 10 L/min, and gas temp: 320 °C.

### 3.6. Statistical Analysis

The data were processed using IBM SPSS Statistics 23.0 (IBM Corp., New York, NY, USA) and visualized with GraphPad Prism 10.4.1 (GraphPad Software, San Diego, CA, USA). Continuous variables are expressed as mean ± standard deviation (SD) for normally distributed data (verified by a Shapiro–Wilk test) or median (interquartile range) [M (IQR)] for non-normally distributed data; the between-group homogeneity of variance was confirmed with Levene’s test. For two independent groups with a normal distribution and equal variances, independent-sample *t*-tests were applied. Paired measurements meeting normality assumptions used paired *t*-tests. Non-normally distributed unpaired data underwent Mann–Whitney U testing, while paired non-normal data used Wilcoxon signed-rank tests. Categorical data were analyzed by the χ^2^ test. Statistical significance was defined as *p* < 0.05.

## 4. Results

### 4.1. KMO Inhibition Reduces Seizure Frequency and Severity in Epileptic Mice

**Figure 3:**
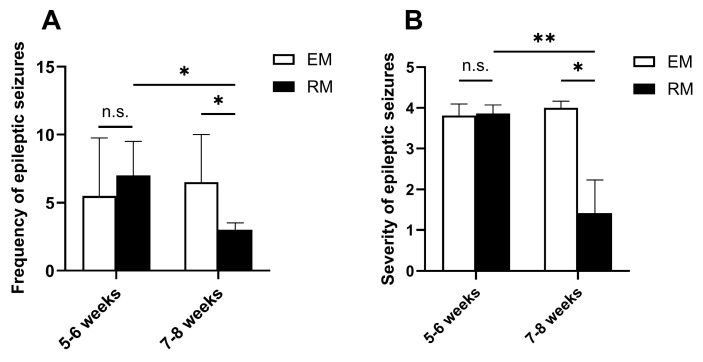
Ro 61-8048 reduces seizure frequency and severity in epileptic mice. (**A**) Seizure frequency pre- vs. post-intervention. No significant between-group difference pre-intervention (*p* > 0.05). Post-intervention: RM group showed significant reduction vs. baseline (*p* < 0.05), while EM group showed no change. (**B**) Seizure severity scores pre- vs. post-intervention. Comparable baseline severity between groups. Post-intervention: RM group exhibited significantly decreased severity vs. baseline (*p* < 0.05) with no change in EM controls. Data represent median (IQR) (*n* = 6 mice/group). Within-group comparisons: Wilcoxon signed-rank test; and between-group comparisons: Mann–Whitney U test. * *p* < 0.05, ** *p* < 0.01, and n.s. = not significant. EM: epileptic model group; and RM: Ro61-8048-treated epileptic model group.

A baseline analysis revealed no statistically significant differences in seizure frequency or severity between the EM and RM groups pre-intervention (*p* > 0.05). Post-intervention video monitoring demonstrated that saline-treated EM controls exhibited no significant changes in seizure frequency [pre: 5.5 (3.75–9.75) vs. post: 6.5 (4.25–10); *p* > 0.05] or severity [pre: 3.82 (3.73–4.10) vs. post: 4.00 (3.76–4.16); *p* > 0.05]. Conversely, Ro 61-8048-treated RM mice showed significant reductions in both frequency [pre: 7.0 (3.75–9.5) vs. post: 3.0 (1.5–3.5); *p* < 0.05] and severity [pre: 3.87 (3.73–4.07) vs. post: 1.42 (1.00–2.23); *p* < 0.05]. These results indicate that Ro 61-8048 can reduce seizure frequency and significantly alleviate seizure severity in epileptic mice. The relevant data are presented in Figure 3A,B.

### 4.2. Body Weight Dynamics

**Figure 4:**
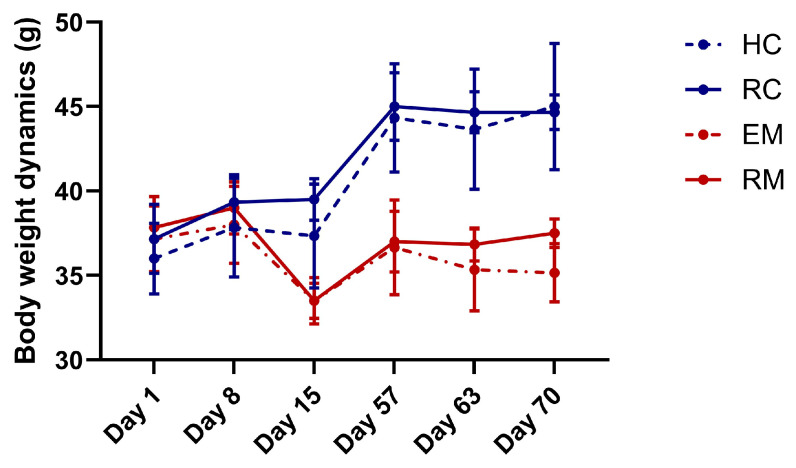
Body weight dynamics in experimental groups. Ro 61-8048 attenuated seizure-induced weight deficits and restored physiological growth in epileptic mice without causing pathological weight deviations. Data are presented as mean ± SD (*n* = 6 mice/group). HC: Healthy control group; RC: Ro61-8048-treated healthy group; EM: epileptic model group; and RM: Ro61-8048-treated epileptic model group.

The dynamic changes in body weight across groups are presented in Figure 4. At baseline (Day 1), no significant differences occurred among HC (36.00 ± 2.10 g), RC (37.17 ± 2.04 g), EM (37.17 ± 1.94 g), and RM (37.83 ± 1.83 g) (*p* > 0.05), confirming the comparable group allocation. After one week of environmental adaptation, both HC and RC exhibited significant weight increases (*p* < 0.05), indicating successful acclimation without environmental stress interference. Following pilocarpine-induced seizures (Day 7), EM and RM demonstrated significantly lower weights than HC/RC (EM: 33.50 ± 1.22 g; RM: 33.50 ± 1.05 g; *p* < 0.05), reflecting a seizure-related physiological compromise. No significant weight differences existed between EM and RM (*p* > 0.05). At endpoint (Day 70), RM showed significant weight recovery (37.33 ± 0.82 g) compared to its post-seizure baseline (33.50 ± 1.05 g; *p* < 0.05), exceeding EM (35.17 ± 1.72 g; *p* < 0.05), though remaining below RC levels (44.67 ± 0.99 g; *p* < 0.05). These findings indicate Ro 61-8048 promotes recovery from a seizure-induced weight deficit without causing pathological abnormalities.

### 4.3. KMO Inhibition Improves Depression-like Behaviors in Epileptic Mice

**Figure 5:**
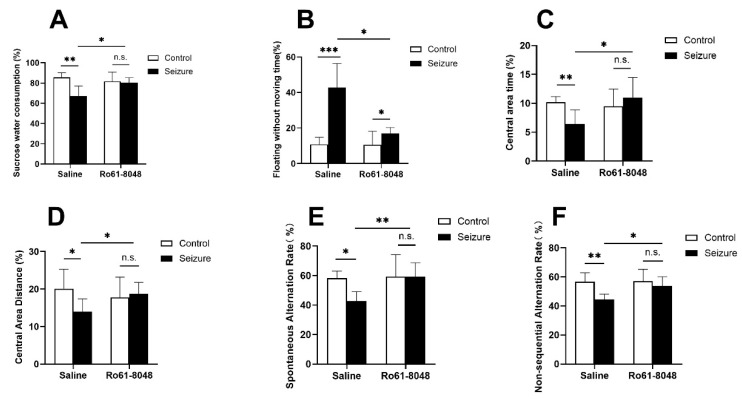
Ro 61-8048 alleviates epilepsy-induced depressive-like behavior and improves cognitive function in epileptic mice. Ro 61-8048-treated epileptic mice exhibited the following: (**A**) significantly increased sucrose preference in the sucrose preference test; (**B**) reduced immobility time in the forced swim test; (**C**) increased movement distance in the central zone of the open field test; (**D**) prolonged duration in the central zone of the open field test; (**E**) increased spontaneous alternation rate in the Y-maze test; and (**F**) reduced non-sequential alternation rate in the Y-maze test. Data were presented as mean ± SD (*n* = 3−6 mice/group). Histograms represent statistical differences between groups. * *p* < 0.05, ** *p* < 0.01, *** *p* < 0.001, and n.s. = not significant.

In the SPT, the percentage of sucrose preference in the EM group was significantly lower than that in the HC group (*p* < 0.05). Conversely, RM showed a significantly higher preference than the EM group (*p* < 0.05), and not statistically different from that in the RC group (*p* > 0.05) (Figure 5A), indicating a reversal of epilepsy-induced anhedonia by Ro 61-8048.

In the FST, EM displayed a significantly longer immobility time than HC (*p* < 0.05). RM significantly shortened the immobility time versus EM (*p* < 0.05), though it remained elevated relative to RC (*p* < 0.05) (Figure 5B), suggesting the partial amelioration of depression-like behaviors.

### 4.4. KMO Inhibition Does Not Exacerbate Cognitive Impairment in Epileptic Mice

In the OFT, the EM group showed a significantly reduced center time versus HC (*p* < 0.05). After the Ro 61-8048 intervention, RM exhibited a significantly increased center time versus EM (*p* < 0.05), with no statistical difference from RC (*p* > 0.05) (Figure 5C). Similarly, the center distance traveled was significantly lower in EM versus HC (*p* < 0.05), but significantly increased in RM versus EM (*p* < 0.05) without any statistical difference from RC (*p* > 0.05) (Figure 5D), indicating improved exploratory behavior.

In the Y-maze, the spontaneous alternation rate was significantly reduced in EM versus HC (*p* < 0.05), but elevated in RM versus EM (*p* < 0.05) to levels statistically indistinguishable from RC (*p* > 0.05) (Figure 5E). Conversely, the non-sequential alternation rate was significantly higher in EM versus HC (*p* < 0.05), but reduced in RM versus EM (*p* < 0.05) with no statistical difference versus RC (*p* > 0.05) (Figure 5F), demonstrating the amelioration of epilepsy-induced spatial working memory deficits.

### 4.5. KMO Inhibition Increased KYNA Levels and the KYNA/KYN Ratio, While Decreasing the 3-HK/KYN Ratio and 3-HANA Levels

**Figure 6:**
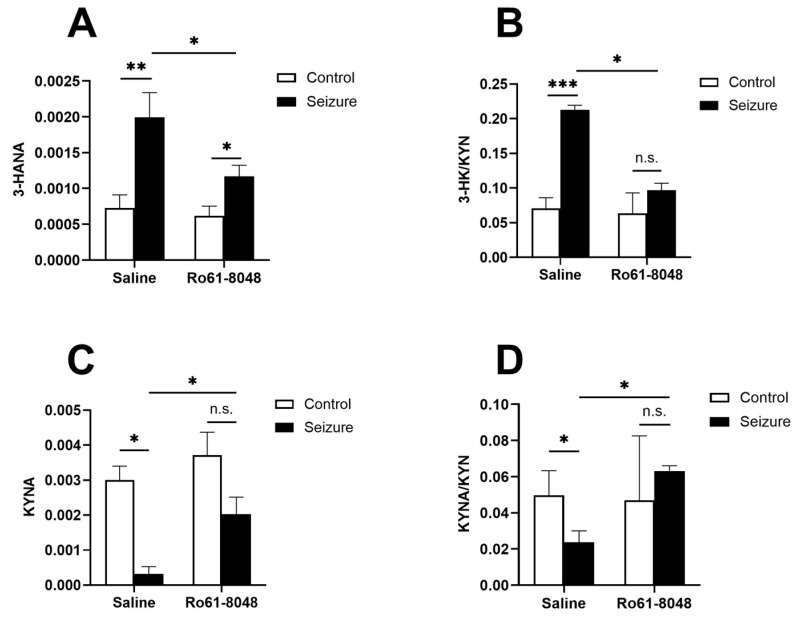
Ro 61-8048 inhibits KMO activation and reversal of the kynurenine metabolic pathway to the neuroprotective branch in epilepsy mice. After Ro 61-8048 intervention, we have the following: (**A**) decreased 3-HANA level; (**B**) reduced 3-HK/KYN ratio; (**C**) increased KYNA level; and (**D**) increased KYNA/KYN ratio. Data represent means ± SD (*n* = 3−6 mice/group). Bars indicate statistical differences among groups. * *p* < 0.05, ** *p* < 0.01, *** *p* < 0.001, and n.s. = not significant.

In the liquid chromatography–tandem mass spectrometry (LC–MS/MS), the 3-HANA levels in the hippocampus of the EM group were significantly elevated compared with the HC group (*p* < 0.05), whereas their levels were significantly reduced after the Ro 61-8048 intervention (*p* < 0.05), but were still slightly higher than those of the RC group (*p* > 0.05) (Figure 6A). Meanwhile, the 3-HK/KYN ratio was significantly increased in the EM group compared with the HC group (*p* < 0.05), suggesting that chronic seizures can activate KMO and promote the production of neurotoxic metabolites, whereas this ratio was significantly decreased after the Ro 61-8048 intervention (*p* < 0.05), and there was no significant difference when compared with the RC group (*p* < 0.05) (Figure 6B).

In addition, the KYNA levels were significantly lower in the EM group compared with the HC group (*p* < 0.05), and their levels significantly rebounded after the Ro 61-8048 intervention (*p* < 0.05), and there was no statistically significant difference compared with the RC group (*p* > 0.05) (Figure 6C). The KYNA/KYN ratio was significantly lower in the EM group (*p* < 0.05), and significantly higher in the post-intervention RM group (*p* < 0.05) and not statistically different from the RC group (*p* > 0.05) (Figure 6D), suggesting that epilepsy leads to the impairment of neuroprotective metabolic pathways, and that the inhibition of KMO can effectively reverse this pathological change.

## 5. Discussion

### 5.1. Main Findings

In this study, the specific KMO inhibitor Ro 61-8048 was administered to epileptic model mice. The results confirmed that KMO inhibition exerts significant dual therapeutic effects: it effectively alleviates seizure severity and improves depressive-like behaviors, without exacerbating cognitive impairment—conversely, it confers a certain improvement in cognitive performance. Additionally, Ro 61-8048 also significantly promotes the recovery of body weight in epileptic mice, indicating its positive impact on overall physiological health. The core mechanism involves correcting the imbalance in the kynurenine metabolic pathway by regulating its metabolic flux, specifically reducing the accumulation of neurotoxic metabolites such as 3-HK and 3-HANA, while upregulating the level of neuroprotective KYNA to the physiological normal range. These findings provide key experimental evidence for KMO inhibitors as a novel targeted intervention strategy for treating epilepsy-associated depression.

### 5.2. Similarities and Differences with Previous Studies

The key innovation of this study is the clear and systematic demonstration that inhibiting KMO activity significantly ameliorates the core behavioral symptoms of epilepsy-associated depression (e.g., anhedonia and behavioral despair) and cognitive dysfunction. Notably, no cognitive deterioration was observed despite elevated KYNA levels following KMO inhibition, which contrasts with the findings from other specific models: In a study by Sophie Imbeault et al., mice with KMO knockout exhibited increased brain KYNA levels and showed deficits in spatial working memory [38]. Similarly, Snezana Milosavljevic et al. used a transgenic KMO mouse model to simulate reduced KMO activity and elevated KYNA, and the results revealed cognitive impairment in the mice [39]. In these models, KMO inhibition-induced KYNA elevation was consistently associated with cognitive deficits. Additionally, previous studies have mostly focused on the neuroprotective mechanisms of Ro 61-8048 [40,41]. The present study, on the other hand, provides the first comprehensive confirmation in a chronic epilepsy model that Ro 61-8048 not only controls seizures, but also significantly improves epilepsy-associated co-morbid depression-like behaviors and cognitive impairments through a multidimensional assessment (seizures, depression, and cognition), providing evidence on the more comprehensive therapeutic potential of this drug. The present study reconfirms that the inhibition of KMO activation is effective in reducing seizure frequency, confirming the same antiepileptic effect that occurs with the inhibition of KMO activation as in previous studies [42].

### 5.3. Main Mechanisms

Ro 61-8048 effectively reduces epileptic seizures and alleviates comorbid depression. Its mechanism is linked to the hyperactivity in the kynurenine pathway induced by chronic epilepsy, specifically through the enzyme KMO. This hyperactivity causes a pathological shift towards the neurotoxic branch of metabolism. As a specific inhibitor, Ro 61-8048 targets this shift, thereby ameliorating the neurochemical environment related to depression.

The biological effects of the kynurenine pathway are profoundly influenced by the activity of enzymes in its downstream branches. KMO serves as the critical enzyme at the pivotal juncture regulating the metabolism of KYN [43]. KYN, the primary metabolite of tryptophan, catalyzed by indoleamine 2,3-dioxygenase (IDO) and tryptophan 2,3-dioxygenase (TDO), is processed into two metabolically divergent branches that generate compounds with opposing neuroactive properties: (1) the neurotoxic branch, where KMO catalyzes the conversion of KYN into 3-HK, 3-HANA, and QUIN; and (2) the neuroprotective branch, where kynurenine aminotransferase (KAT) converts KYN into KYNA. The 3-HK/KYN ratio is a specific indicator reflecting KMO catalytic activity. An increase in the 3-HK/KYN ratio indicates an enhancement in the neurotoxic branch, while a decrease in the KYNA/KYN ratio suggests a suppression of the protective branch [44]. Our studies demonstrate that, in epileptic mice, the 3-HK/KYN ratio is increased while the KYNA/KYN ratio is decreased, confirming the activation of KMO. This marked shift toward the neurotoxic branch forms the critical pathological basis for comorbid depression in epilepsy.

Ro 61-8048 inhibits the conversion of KYN to 3-HK and 3-HANA by directly suppressing the activity of the KMO enzyme [26]. Meanwhile, it promotes KYNA synthesis by diverting the tryptophan metabolic flux toward the neuroprotective branch [45]. As an endogenous antagonist of glutamate (Glu) receptors, KYNA exerts a direct inhibitory effect on the excessive excitation of the glutamatergic system [46], which represents one of the core pathological mechanisms underlying epilepsy [47]. Consequently, Ro 61-8048 reduces toxic metabolites and interrupts the glutamate excitation cycle associated with epileptiform discharges, thereby alleviating the severity of seizures. Although abnormally high levels of KYNA could impair cognitive functions [48], our study observed that the specific dosage and treatment duration of Ro 61-8048 did not significantly increase the KYNA levels. Cognitive improvements were demonstrated in the open field and Y-maze tests, indicating that Ro 61-8048 finely regulates KYNA levels, improving both seizure and depressive behaviors while protecting cognitive functions. This likely results from our choice of appropriate dosages and treatment durations with Ro 61-8048, which prevents the excessive accumulation of KYNA and reduces the risk of cognitive impairment.

3-HK, as an endogenous neurotoxin, leads to the production of a substantial amount of reactive oxygen species (ROS) during its metabolism [49]. This oxidative stress damages synaptic proteins such as PSD-95, disrupting the synaptic structure and function [50], which exacerbates epilepsy-associated depression. Furthermore, elevated levels of 3-HK are associated with neuronal damage, functional impairments, and abnormal electroencephalographic discharges [51,52]. 3-HANA, another oxidative stress metabolite, can accumulate and activate the NLRP3 inflammasome in microglia, promoting the release of inflammatory cytokines IL-1β and Interleukin-18(IL-18) [53]. This inflammatory milieu not only intensifies epileptic seizures but also facilitates the synthesis of the convulsant QUIN, which further activates the microglia to release inflammatory factors [54] and promotes the transcription of the KMO gene [55], thereby creating a vicious cycle of neurotoxicity.

A reduced KYNA/KYN ratio is often associated with increased neuroinflammation and susceptibility to epilepsy [56]. As an endogenous neuroprotective agent, KYNA primarily functions by binding to the glycine site of NMDA receptors, inhibiting the excessive excitation of the glutamatergic system [57]. In states of epilepsy, excessive glutamatergic excitation overstimulates NMDA receptors, leading to neurotoxicity; a decrease in KYNA levels can further exacerbate this pathological process [58]. Ro 61-8048 elevates brain KYNA levels to mitigate neurotoxicity [25], consistent with our research findings. Moreover, KYNA also directly participates in the anti-depressant mechanism. Studies have shown that an intracerebral injection of KYNA significantly reduces immobility time in the forced swim test in male mice. The anti-depressant effects of KYNA can be blocked by pre-treatment with 5-HT2 receptor antagonists and Gamma-Aminobutyric Acid Type A(GABAA) receptor antagonists, suggesting that KYNA exerts its anti-depressant effects by modulating serotonin 2 (5-HT2) and GABAA receptors [59].

### 5.4. Significance of the Study

The present study establishes the centrality of KMO-mediated toxic branching off of the kynurenine metabolic pathway in epileptic co-morbid depression. By demonstrating that the KMO inhibitor Ro 61-8048 can simultaneously reduce seizures and improve depressive-like behaviors without exacerbating cognitive impairment, it reveals, for the first time, that targeting a single metabolic node can synergistically modulate neural excitability and mood loop dysfunction, providing a novel perspective on the mechanism of co-morbidity. This finding not only indicates that the metabolic imbalance of the kynurenine metabolic pathway (the accumulation of toxic products and depletion of protective substances) is a key pathological bridge to reducing the risk of cognitive inhibition in epilepsy-depression co-morbidities, but also suggests that the KMO could be a “dual-action” target, which could be the basis for the development of innovative therapies with both antiepileptic and anti-depressant effects.

### 5.5. Shortcomings of the Study

Although this study has made significant progress in revealing the potential therapeutic effects of KMO inhibitors on epilepsy-associated depression, it has some limitations. Firstly, the study relied solely on a single animal model, specifically, a mouse model of pilocarpine hydrochloride-induced epilepsy, to demonstrate that inhibiting changes in the kynurenine metabolic pathway following KMO activation is associated with the amelioration of seizures, depressive-like behaviors, and cognitive impairment. Further clinical validation is necessary. Additionally, this study focused on the short-term effects of KMO inhibitors. Their long-term therapeutic effects and potential side effects still require a more comprehensive investigation.

### 5.6. Directions for Future Research

Future studies should consider (1) validating the efficacy of KMO inhibitors in a broader range of epilepsy animal models, such as genetic epilepsy models or post-traumatic epilepsy models, to better represent the diversity of human epilepsies; (2) conducting long-term therapeutic studies to assess the long-term efficacy and safety of KMO inhibitors in models of chronic epilepsy and co-morbid depression, and to explore their potential impacts on the disease process; and (3) undertaking clinical validation studies to investigate the efficacy and safety of KMO inhibitors in human patients with epilepsy and co-morbid depression. This will facilitate the translation of basic research findings into clinical practice and provide more effective treatment options for patients.

## 6. Conclusions

The present study confirmed the significant antiepileptic and anti-depressant effects of the KMO inhibitor Ro 61-8048 in an epileptic mouse model, without aggravating cognitive impairment. This finding not only reveals the important role of KMO in comorbid depression associated with epilepsy but also provides strong theoretical support for the development of novel therapeutic strategies. Future studies will further explore the long-term efficacy and safety of KMO inhibitors across various epilepsy models and delve into their multi-targeted mechanisms of action on neurotransmitter systems and neuroinflammation, laying the foundation for clinical applications.

## Data Availability

The original contributions presented in this study are included in the article. Further inquiries can be directed to the corresponding author.
